# The co-creation of Foot Selfie, a patient-centered mobile intervention to prevent diabetic foot ulcers

**DOI:** 10.1186/s12913-026-14718-5

**Published:** 2026-06-17

**Authors:** Guillermo Almeida-Huanca, Josselyn Jauregui, David Bustos, Milagros M. Quispe-Mendoza, Dayana Ticona, Lourdes Cruzado-Castro, María Lazo-Porras, J. Jaime Miranda, Pablo Perel, Juned Siddique, Jacqueline Seiglie, Hueiming Liu, Laura E. Downey, David Beran, Germán Comina, David G. Armstrong, Stephen Jan, Sigiriya Aebischer Perone, Jessica Hanae Zafra-Tanaka

**Affiliations:** 1 CRONICAS Center of Excellence in Chronic Diseases, Universidad Peruana Cayetano Heredia, Lima, Peru; 2 Research Group in Engineering for Health and the Environment (SAMA-UNI), Facultad de Ciencias, Universidad Nacional de Ingeniería, Lima, Peru; 3 Sydney School of Public Health, Faculty of Medicine and Health, The University of Sydney, Camperdown, NSW Australia; 4 Faculty of Epidemiology and Population Health, London School of Hygiene & Tropical Medicine, London, UK; 5 Department of Preventive Medicine, Northwestern University Feinberg School of Medicine, Chicago, USA; 6 Department of Medicine, Massachusetts General Hospital, Boston, MA USA; 7 The George Institute for Global Health, University of New South Wales, Sidney, Australia; 8 The George Institute for Global Health, Imperial College London, London, UK; 9 Division of Tropical and Humanitarian Medicine, Faculty of Medicine, University of Geneva and Geneva University Hospitals, Geneva, Switzerland; 10 Department of Surgery, Keck School of Medicine of University of Southern California, Los Angeles, USA

**Keywords:** Patient and public involvement, Co-creation, Diabetic foot ulcers, Meaningful engagement

## Abstract

**Background:**

Diabetic foot ulcers (DFU) represent a major global burden, requiring more effective prevention strategies. Digital solutions have been proposed to improve DFU early-detection and reduce DFU burden, but user-centered solutions for low-and-middle income countries (LMIC) are lacking. The aim of this paper is to describe the process and lessons learned from the co-creation of (1) an intervention based on a mobile health (mHealth) app for early detection of pre-ulcerative lesions and prevention of DFU in people living with diabetes, and (2) the strategy to pilot test the intervention in rural and urban settings in Peru.

**Methods:**

We conducted a co-creation process between December 2024 and April 2025. Four workshops were held in Lima (urban) and four in Piura (semi-rural), involving 54 people living with diabetes, their caregivers, and healthcare professionals (HCPs). In workshop #1, patient journey mapping was used to identify barriers and facilitators to DFU care. Workshops #2 and #3 focused on the co-development of a mHealth app, while workshop #4 gathered participant feedback on study procedures for the upcoming pilot study.

**Results:**

A total of 23 people with diabetes, 21 HCPs, and 10 caregivers participated in the workshops. Patient journeys revealed barriers to DFU management, including health system fragmentation, limited clinician training, and insufficient education for people with diabetes and caregivers. In workshops #2 and #3 a beta version of the mHealth app was tested by the participants who proposed recommendations on how to improve it and other features to be added. Study procedures for the coming pilot study of the developed intervention were tested with role play. Participants recommended community-based recruitment of participants for the pilot study, manuals and videos to train users, family engagement, WhatsApp reminders, a helpline, interim feedback calls, and graphical results in multiple formats. Participant engagement was sustained throughout the study, with participants contributing from barrier identification through to intervention and procedure design.

**Conclusions:**

This work describes the usage of a structured co-creation processes to develop a digital health tool for DFU prevention and detection. The co-creation process was conducted with the aim to develop an intervention that is contextually relevant, acceptable, and more likely to be implemented successfully in the Peruvian context. The processes described here offer a practical model for developing DFU-prevention interventions in similar resource-constrained environments. The upcoming pilot study will be essential to evaluate usability, adherence, and early signals of impact, ultimately guiding future scale-up.

**Supplementary Information:**

The online version contains supplementary material available at https://doi.org/10.1186/s12913-026-14718-5.

## Background

Co-creation is a collaborative process in which different stakeholders work together from the identification of a problem to the design of solutions centered in the end-user, creating shared benefits [1]. Engaging in co-creation improves cultural pertinence and the buy-in of people with lived experience [2]. The umbrella of co-creation includes co-design, a tool that focuses on the development of specific solutions with the participation of end-users. Co-design can achieve the development of solutions that meet the needs of the end-users and place their expertise and knowledge at the center [1].

Co-creation in health research can help ensure that the development on health interventions and their assessment are in line with end-users’ needs and expectations. We conducted a co-creation process to develop an intervention for the prevention of Diabetic foot ulcers (DFU) in low-and-middle income countries (LMIC) with the participation of people living with diabetes, caregivers, and health care professionals (HCPs).

DFU represent one of the most severe, disabling and expensive complications of diabetes [3]. An estimated 20% of people who develop DFU require hospitalization and between 15 and 20% of them will require an amputation [3, 4]. Additionally, people living with diabetes who develop DFU have higher mortality rates than those who do not [5, 6]. Managing DFU in LMICs represents a significant challenge given the weaknesses within the healthcare system, scarcity of trained HCPs in diabetic foot care, and limited availability of wound care services [6]. However, DFU burden on both individuals and the systems in place to support them could be reduced when preventive interventions are in place [7].

Prevention of DFU is an essential part of diabetes care [7]. International guidelines recommend regular inspection of the feet, appropriate footwear, self-management education, and early detection of pre-ulcerative lesions [7]. However, implementing these strategies remains challenging. In LMICs, in comparison to high-income countries (HIC), DFU are diagnosed at more advanced stages leading to higher complications and mortality rates. Thus, innovative strategies to prevent and achieve early identification of DFU in LMIC settings are key to reduce its burden [6].

Digital solutions have shown promise in assisting management of DFU as they offer opportunities to expand access and reach a larger population [8]. Yet, their implementation is lagging globally because of diverse factors such as negative beliefs about technology and confidentiality of data or problems related to usability and adaptability to the end-users, among others. So, it has been suggested that future digital solutions focus on user-friendly and adaptable digital technologies [8, 9]. For these reasons, we decided to conduct a co-creation process to develop a digital intervention for the prevention of DFU in the Peruvian context.

The aim of this paper is to describe the process and lessons learned from the co-creation of: (1) an intervention based on a mobile health (mHealth) app for early detection of pre-ulcerative lesions and prevention of DFU in people living with diabetes, and (2) the strategy to pilot test the intervention in semi-rural and urban settings in Peru.

## Methods

### Design

We adopted a co-design approach, understood here as a form of co-creation in which active collaboration between stakeholders facilitates designing solutions to a prespecified problem [1]. Specifically, we conducted co-creation workshops between December 2024 and April 2025 with the following aims: To (1) identify barriers to DFU care, (2) co-develop an mHealth app-based intervention, and (3) shape study procedures for a future pilot study of the intervention. Four workshops were held in Lima (urban) and four in Piura (semi-urban) with people living with diabetes, their caregivers, and HCPs. In conducting these workshops and following NIHR principles of Patient and Public Involvement (PPI), defined as research conducted with or by members of the public rather than to, about or for them, our primary objective was to strengthen the relevance, quality and impact of the proposed mhealth DFU intervention [10]. The activities followed the National Institute for Health and Care Research from the UK (NIHR) proposed principles for co-production of research, namely: power sharing, including all perspectives, respect & valuing others, reciprocity, and building relationships [11].

### Context

The activities reported in this paper are part of the FootSelfie project which aims to develop and test an innovative approach for the prevention of DFU [12]. The development of the intervention started with formative research to identify features of an ideal mHealth app aimed at preventing DFU. A draft beta version of the app was used as a stimulus for guiding the co-creation of the first version of the app. For this paper, we focus only on the co-creation workshops in which the beta version of an app was modified according to the input of people with diabetes, caregivers and HCPs. The activities that lead to the development of the initial version of the app have been described in detail in Additional file 1.

### Setting

**Fig. 1:**
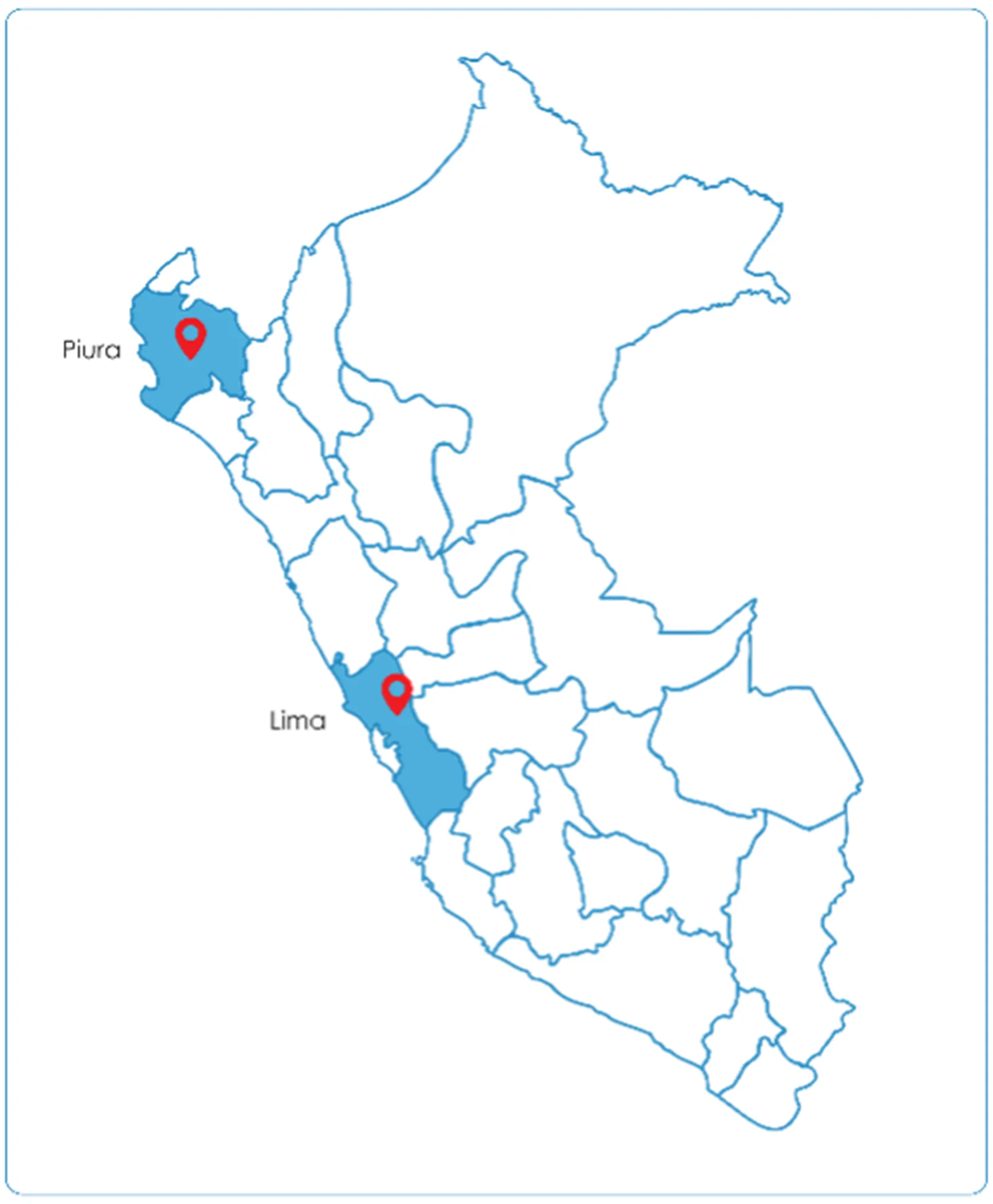
Map of study sites

Lima is the capital of Peru and an urban area with the greatest access to specialized health services. Piura, a region in the north of the country, has one of the lowest densities of services and a high prevalence of complications associated with diabetes. These locations were selected due to their significant diabetes burden [13], and because of the level of urbanization: Lima an urban city and Tambo Grande, Piura semi-urban (Fig. 1).

### Participants

Three different stakeholder groups participated in the workshops: people living with diabetes, caregivers of people living with diabetes, and HCPs. The inclusion criteria were as follows: adults living with diabetes (≥18 years of age) undergoing treatment for diabetes at a public facility at high risk of DFU (either with history of DFU or amputation); family caregivers without professional health training; and HCPs consisting of doctors and nurses with experience in diabetic foot care.

We used convenience sampling to identify participants through multiple channels: selection from a list of participants of a previous study within the same project (WP1 of the FootSelfie project, see Additional files for details), referral by other participants, or identification by HCPs who were participating on other components of the FootSelfie project. Potential participants were contacted by telephone, to explain the objectives of the project, the location, and the dates of the workshops. The recruitment goal was two participants per profile (people at high risk of DFU (history of DFU or amputation), caregivers, nurses, and doctors) to enroll 10 participants per region.

All the stakeholders informed three different stages of the research process: (1) identifying barriers to DFU care, (2) co-developing the mobile intervention, and (3) shaping study procedures for a future pilot study of the co-created intervention. The level of involvement was active and collaborative. Participants engaged in different activities, evaluated prototypes, recommended methodological adaptations to the study procedures, and provided feedback according to their needs.

### Study procedures

We planned the co‑design process into three stages, delivered across four workshops in each site. The workshops were structured following an iterative process of planning, acting, observing, and reflecting, with the aim of ensuring constant improvements of the workshops’ content and structure based on direct experience.

Stage 1: Understanding current DFU care and identifying barriers (Workshops 1 and 2). In workshop 1, we used patient journey maps developed in Work Package 1 of the FootSelfie project (unpublished) to structure a group discussion about barriers and facilitators to accessing DFU care. Participants reviewed and amended the journey maps and jointly identified key barriers along the care pathway.

Stage 2: Co-designing the intervention by generating and testing ideas (Workshops 2 and 3). The barriers identified in workshop 1 were taken forward to workshops 2 and 3, where participants explored whether these could be addressed through an mHealth app. In these workshops, participants tested two iterative versions of the app and provided feedback on content, usability and additional features. Between workshops, the engineering team incorporated the suggested changes into updated prototypes for further testing.

Stage 3: Refining pilot study procedures (Workshop 4). In workshop 4, participants engaged in role‑playing activities to help define and refine procedures for a future pilot study, including recruitment, consent, use of the app in routine care, and follow‑up.

Stakeholder suggestions from each workshop were collated and discussed by the research team, and were incorporated whenever they were judged to be safe, feasible within available resources, and consistent with the project scope; where this was not possible, we adapted proposals in consultation with participants in subsequent workshops. See Table 1 for more details.

**Table 1:** Description of the workshop activities

Workshop	Aim of the workshop	Activities / Outcomes
#1	To map the journey of a person living with diabetes in terms of diabetic foot care flows within the public health system of Peru	- Review and validation of the Patient Journey. - Group discussion: analysis of barriers and facilitators to access care, and potential solution
#2	To present the beta version of the mHealth app, which integrates the previously trained artificial intelligence model to properly identify pre-ulcerative lesions in foot photographs	- Group discussion to prioritize moments of the patient journey - Explore the user experience of using the app - Exploring the motivation to use app - Synthesis and integration of solutions within the patient journey
#3	To present the new version of the mHealth app, which integrates the previously trained artificial intelligence model to properly identify pre-ulcerative lesions in foot photographs	- Exploring the usage of app at home using the COM-B model (capacity, opportunity, motivation) - Brainstorming recommendations and suggestions for the FAQs developed - Explore the user experience of using the app - Validation of educational material
#4	To identify, using role playing technique, the personal, community, and institutional factors that could facilitate the conduction of a pilot study to test the co-developed intervention	- Exploring the usage of app at home - Explore the user experience of using the app - Work on naming of the app - Identify best practices for the pilot study procedures - Validation of materials (e.g. planner) - Validation of the way results are presented

The workshops lasted approximately half a day and were held mainly on weekends, except for two workshops in Piura which were organized on weekdays. The dates of the co-creation workshops were coordinated with participants to avoid scheduling conflicts.

### Study team

The co-creation workshops were facilitated by an interdisciplinary team of professionals with backgrounds in sociology, anthropology, economics, psychology, medicine, and nursing. In Lima, facilitators included a psychologist, two anthropologists, three app developers, two doctors, and two nurses. In Piura, two psychologists, a nurse, a technology specialist, and an economist facilitated the workshops. Additionally, reporters took notes of the discussions following a structured format based on the workshops’ aims. Three reporters participated in Lima, and two in Piura. Supervision of the workshop activities was done by one member of the research team: one nurse and one anthropologist with experience in fieldwork and co-creation methodologies. In addition, the research team was available to provide logistical support.

The facilitators and reporters participated in training sessions that included reviewing technical content on diabetes, facilitation dynamics, and activity simulations. These sessions allowed for reflection on possible biases, ensured respectful interaction, and standardized the criteria for conducting and reporting on the workshops prior to implementation. A workshop guideline including the aims of the workshop, activities to be conducted, times and reporting guides was developed to facilitate the training sessions. The guideline was also available for consultation during the workshops.

The facilitators and reporters had no prior relationship with the participants. No outsiders were present at the workshops, except for the research team and the invited participants.

### Data collection

Facilitators and reporters were asked to fill a report for each workshop which included a description of the participants; a synthesis of the discussions during each activity; a summary of users’ experiences with the application; and an observational record of participant interactions throughout the workshop, including levels of collaboration, moments of conflict, and the strategies used to resolve them.

### Data analysis

A synthesis of the information collected during each workshop was carried out by the study team and used to adjust subsequent sessions. For this paper, we extracted information from the workshop reports into an Excel sheet. The following information was extracted: workshop number, location, description of participants and facilitators, duration (in hours), workshop objectives, techniques employed, outputs generated in each workshop, and lessons learned regarding the methodology used, the health system, and the pilot implementation phase. The analysis was descriptive. Initially, the lessons learnt were listed and similar items were grouped under broad descriptive categories. As an example, to identify the barriers to care, we identified the issues mentioned across groups and workshops (e.g., “insufficient training leading to delays in treatment and diagnosis”, “education on DFU prevention provided to the patient is limited”, etc.), to then consolidate similar topics into one (e.g., “need to improve training for HCPs”). The same procedure was then followed for other topics such as the methodology used or the implementation phase. Two authors worked on this process, and discussions were held with a third author to decide the final categories. The resulting synthesis was then reviewed and validated by the rest of the study team.

### Ethics

We received approval from the pertinent IRBs; Universidad Peruana Cayetano Heredia with institutional code (IRB: CIEI-E-045-05-25) and the London School of Hygiene & Tropical Medicine (Ref:30662) to conduct the study, and all participants signed an informed consent form agreeing to be part of the study. The sessions were designed to respect the cognitive, cultural, and emotional capacities of the participants, ensuring a safe and respectful environment.

## Results

A total of 50 people agreed to participate, 35 in Lima and 15 in Piura. In Lima, 11 patients undergoing treatment for DFU, two patients who underwent amputation, seven caregivers, six nurses, and nine doctors participated. In Piura, there were five patients undergoing treatment for DFU, two patients who underwent amputation, three caregivers, three nurses, and two doctors.

Participant retention in the co-creation workshops was high. In Lima, 25 of the 35 enrolled participants (71.4%) attended all workshop sessions, while in Piura, 13 of 19 participants (68.4%) completed all sessions. Absences were mainly due to conflicting activities, medical appointments, or difficulties traveling from home to the workshop venue.

Participants in Lima ranged in age from 24 to 72 years, while those in Piura ranged from 24 to 76 years. Most of the participants were female 68.6% in Lima and 63.2% in Piura. Characteristics of the workshop participants are reported in Table 2.

**Table 2:** Characteristics of the cocreation workshop participants

Characteristic	Lima (*n* = 35)	Piura (*n* = 19)
**Age range (years)**	24–72	24–76
**Participant profile**		
Participants with a history of DFU	4	4
Participants who underwent amputation	9	6
Caregivers	7	3
Healthcare providers	15	6
**Sex**		
Female	24	12
Male	11	7

### Co-creation workshops

The co-design was planned in three stages: (1) Understanding current DFU care and identifying barriers (Workshops 1 and 2), (2) Co-designing the intervention by generating and testing ideas (Workshops 2–3), and (3) Refining pilot study procedures (Workshop 4). Below, we describe the key findings and related outcomes from each of these workshops.

Stage 1: Understanding current DFU care and identifying barriers (Workshops 1 and 2).

Participants discussed the patient journey maps and prioritized the most important issues within the healthcare system: health system organization, training of healthcare workers, and patient education.

Some patient-reported challenges related to the organization of the healthcare system were delayed diagnosis of diabetes complications, limited medical appointments, insufficient coordination of care between different HCPs and the different levels of care, and lack of clinical practice guidelines for the management of DFU. People living with diabetes responded to these challenges through peer support. This type of support helped newcomers navigate the system and get the emotional support they needed to accept the condition and adhere to treatment.

Participants also reported that insufficient training in DFU of HCPs that work with people with diabetes represents a major barrier, leading to inadequate care and delayed interventions. Participants suggested that continuous education in prevention, early detection, and effective communication strengthens patient trust and adherence. Certification-backed training programs were also identified as effective incentives to improve care quality and reinforce professional commitment.

Participants also identified the need to improve strategies used to educate patients and involve caregivers. Key aspects of patient education were identified: providing emotional support, clear and accessible information, educational materials and interactive workshops to facilitate understanding of self-care and warning signs.

Stage 2: Co-designing the intervention by generating and testing ideas (Workshops 2–3).

**Fig. 2:**
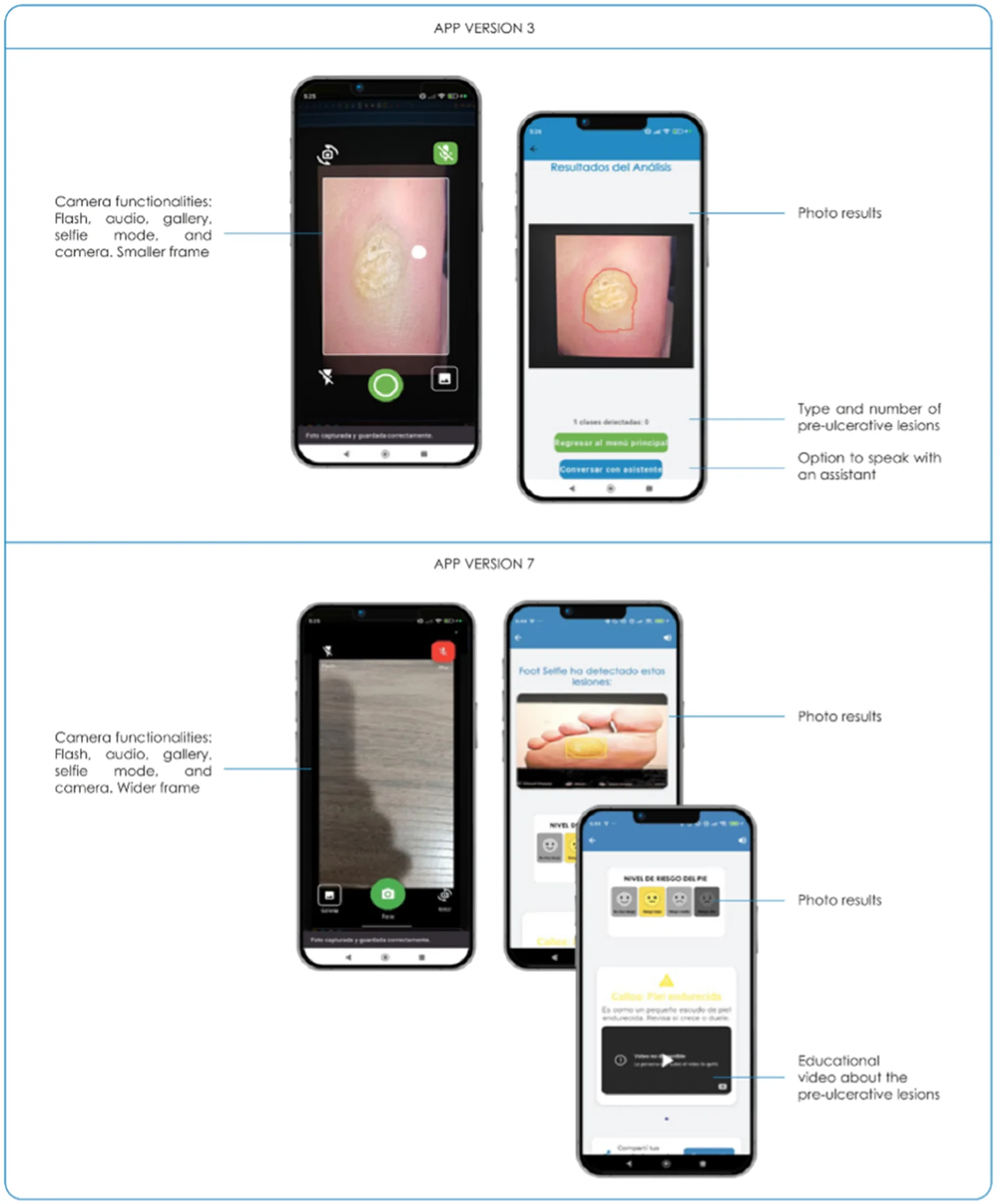
Before and after the co-creation workshops: example of the Camera feature of the mHealth app

Substantial changes were incorporated to the mHealth app-based intervention during the workshops following the discussions with the different end-users. At the beginning of the workshops, the mHealth app consisted of three main sections: ***Home***, which provided information about how to take photos, view the history of results, and check health data; ***Camera***, which allows users to take photos of their feet for tracking purposes; and ***Profile***, where users can edit their personal information. A detailed description of these modules is presented in the additional file 2 which contains a description on the initial version, the last version of the app and the reasons why this was updated (considering the arguments mentioned by the participants and the feasibility aspects that were considered by the study team (Fig. 2).

The Home section was enhanced with instructional videos about how to take photos with the app, a section for frequently asked questions, and a chatbot to provide help regarding the use of the application. This was done following the discussions around doubts on how to use the app that were present in all users.

Both people with diabetes and caregivers suggested modifications to the Camera section to improve its usability, particularly for participants who cannot bend and press a bottom to take the picture of the foot. So, visual guides and voice-activated picture-taking feature was added.

Finally, with the input of people with diabetes and HCPs, the information in the Results section was reorganized to display ulcer risk levels and provide video recommendations tailored to the stage of the lesion, with particular emphasis on pre-ulcerative lesions. In the workshops, people with diabetes were presented with different ways of presenting the results and were asked what actions the messages prompt in them and what emotions the messages would generate. Based on their feedback, messages were better crafted to be able to convey the need to act (e.g. consult a physician) without creating alarm in the patients.

During the co-creation workshops, people living with diabetes expressed the need to access educational material about diabetes and to have reminders to conduct the different activities to prevent DFU. For that reason, a new module called “My Care” was created. This module provides educational resources validated by people with lived experience, practical guides, and personalized configuration options to support self-care such as an alarm and a calendar. Additionally, people with diabetes and caregivers highlighted the importance for being in contact with peers to receive support and the difficulties they faced when accessing healthcare services. Thus, they proposed two directories of diabetic organizations and health centers with diabetic foot units to strengthen connections with local services. This information was included in a new module called “More”.

Some recommendations were not included due to logistical constraints. For example, functionality to communicate with other users of the app and a monitoring dashboard for HCPs were proposed. The possibility of exchanging experiences between users through WhatsApp groups or forums was ruled out due to additional costs and the need for constant moderation, opting instead to offer directories of health centers and patient associations from which participants could get peer support. HCPs suggested having a dashboard to track their patients’ progress. This idea was discarded because the application was primarily intended for patients.

Stage 3: Refining pilot study procedures (Workshop 4).

The procedures were organized in four stages: recruitment, installation of the app, follow-up, and communication of results. See additional file 3 for details.

Recruitment stage: Participants proposed advertising the project in waiting rooms, providing a clear explanation of the study aims and procedures using visual materials, providing education on DFU prevention at enrollment, and offering incentives such as care kits (e.g. bandages, dressings, gauze, suture pack, among others) or financial support (e.g. paying for transportation). After careful consideration, the study team decided that initial contact would be made by healthcare professionals in waiting rooms, as DFU risk staging is required for eligibility and flyers alone would be insufficient. Visual material such as flipcharts, flyers, and an educational video to be used at the recruitment phase to explain study procedures and to provide education on prevention of DFU were developed by the study team based on the suggestions of the workshop participants (e.g. topics to include) and validated with civil society members (see the role of the National Advisory Committee (NAC) in appendix). Incentives were limited to financial support for transportation and delivery of clinical results, as medical supplies require a prescription.

App Installation: Participants recommended assessing the type of cell phone, internet access, tech literacy prior to enrollment, and involving caregivers or family members to support app usage and follow-up. In response, a technology verification checklist and a questionnaire on tech literacy were developed. Additionally, the study procedures now include the identification of a support person when necessary.

Follow-up: Participants recommended providing materials to explain the functionalities of the app, clarifying what happens if users change cell phones, offering technical support, and maintaining communication through reminders and motivational messages. These suggestions were implemented by incorporating an interactive user manual into the app, backing up the information in a cloud database linked to the phone number, and enabling a contact number along with a WhatsApp channel to resolve technical queries. In addition, weekly notifications according to the level of use were incorporated to reinforce the usage of the app.

For the communication of results, participants recommended providing results in both printed and digital formats, including comparative images of the first and last photos, and accompanying the reports with personalized messages recognizing each person’s effort. These new reporting formats will be used in the pilot study.

## Discussion

In this study, people living with diabetes, including some with a history of DFU or amputation, caregivers and HCPs participated in co-creation workshops to develop a mobile health application and to shape study procedures for a future study to pilot the application designed for the early detection of pre-ulcerative lesions and prevention of DFU.

In our study, different co-creation methods were used. ​​Journey mapping revealed barriers and facilitators for DFU care, including challenges in health system organization, limited training among healthcare professionals, and insufficient education and support for people living with diabetes and their caregivers. In addition, these discussions highlighted that diabetes care takes place mainly outside the healthcare system, in the person’s house and community, making social support systems important for an adequate management of diabetes and prevention of DFU. The participation of different stakeholders in the co-design of an app to prevent DFU led to modifications of the initial features and incorporation of new ones based on the different stakeholders’ needs. Finally, study procedures for the pilot study were modified according to the recommendations of the participants. These modifications included, but were not limited to, better recruitment strategies and to provide results and feedback to participants. These discussions have enabled us to better prepare for the pilot study to be conducted in the following months.

In Peru, co-creation has been used previously to develop a complex intervention for the management of non-communicable diseases in rural areas [14, 15]. Using this method allowed the identification of problems and setting of priorities, in addition to facilitating the generation of trust and cultural adequacy [15]. Using co-creation is a way of facilitating PPI in research, ultimately enhancing its relevance, local acceptance, and overall quality.

Although co-creation can offer significant benefits, its implementation can be challenging due to limited resources, low health literacy, and tensions that may arise among the different stakeholders [16]. During the co-creation process, we were able to identify two key aspects that need to be considered when involving different stakeholders such as patients: (a) flexibility in terms of logistics, content, and methods used, and (b) balancing priorities. Flexibility has been identified as a structural requirement to enable effective participation [17]. In this study, flexibility referred to adapting the timing (days and hours) of activities to participants’ availability, as well as adjusting language and workshop dynamics as needed. Regarding timing, previous research has emphasized the importance of accommodating healthcare professionals and decision-makers due to their time constraints [18]. For example, we coordinated the dates at least two weeks in advance and proposed different hours for the workshops conducted in Lima versus those in Piura.

Language and cultural adaptation are key to understanding different activities. Some studies propose using simple messages and using translations to the local language [16] or working with interpreters during the activities [19]. In our study, we decided to use terms commonly used in the region. As an example, instead of saying “callo hemorrágico” (“hemorrhagic callus”), the term “sangre molida” (“mixed blood”) was used to allow for understanding and facilitate participation.

Some studies have suggested different strategies to maintain attention of the participants. These include organizing short sessions [17] or using audiovisual materials to facilitate understanding [20] and capture attention. Moreover, game-like activities and storytelling have been suggested as strategies for participants with lower educational or reading levels [21], or when digital barriers arise [22, 23]. In our case, we conducted different activities (e.g. games or role playing) in the workshops that allowed us to maintain active participation and retain the attention of the participants. Additionally, the activities proposed were adequate for illiterate participants.

Balancing priorities of the different stakeholders’ groups and the research team was a challenge when conducting our study. We identified two main scenarios that were difficult to handle: (a) tensions between the different participants profiles, and (b) differences in expectations of the participants and the project scope. Some tensions between people living with diabetes and HCPs arose when discussing the journey map and the healthcare systems’ functioning. At the beginning, the different participant groups identified the problems and tried to assign blame to the other. As the discussions continued, it was accepted that the issues of the healthcare system were systemic and that collaborative work was needed to move forward. Other studies have faced challenges when involving HCPs, decision makers, and people with lived experience [15, 24]. These challenges can occur when decision makers prioritize their own agenda, making it hard to align with patients’ expectations [24].

Guided by the NIHR principles of power sharing, including all perspectives, and respect and valuing, the facilitators deliberately created space for each of the participants to contribute and sought to share power in the activities (e.g. using small groups, inviting all participants to speak, rotating who reported back, etc.). These strategies allowed for divergent views to surface and prevented that one group dominated the discussions. Identifying these views helped us better understand the issues related to DFU-prevention and the specific need that each stakeholder had in terms of an intervention. Our experience aligns with previous work showing that valuing the different points of view, particularly those of people with lived experience, supports power sharing, active participation, and a sense of ownership of the intervention [25, 26]. Additionally, acknowledging and placing value on the different backgrounds, values and skill sets of the participants, facilitates collaboration [27]. Finally, we found that careful selection of participants (e.g. roles, seniority and lived experience) is also key to balance power between the different stakeholders’ groups [14].

Some tension also arose related to the differences in expectations of the participants and the project scope and limitations, a challenge often observed in LMIC due to limited time, financial resources, and the need to balance scientific rigor with participants’ preferences [28]. As an example, participants had high expectations related to technology and the usage of chatbots to provide information on diabetes care. However, this was not feasible within the timeline of the project and could not be safe without being properly tested. To communicate this, the NIHR principle of building relationships was prioritized. Thus, the study team valued the input provided by the participants, while explaining the technical and safety challenges of creating this feature. Other studies have faced issues related to this; proposals for solutions that were too specific or out of the scope of the project [14], uncertainty about the co-creation process [29], or difficulties communicating technical aspects to participants [30].

### Strengths and limitations

The strengths of our study are the interprofessional teams conformed by professionals with different backgrounds (medicine/clinical, social sciences, and engineering), the engagement of different stakeholders’ groups in several interactive workshops and having the support of a National Advisory Committee (NAC) to guide decisions. These allowed for involvement of people living with diabetes in different aspects of research (identifying problems, co-developing an intervention, and shaping study procedures). In addition, the novelty of the intervention made it attractive to the participants facilitating engagement in the different discussions.

Our study has some limitations. The impact of participation was not directly measured. However, the products developed with the participation of the different stakeholders show how the views of the participants impacted the research outputs. Moreover, the high retention rate for the workshops reflect that participants felt committed to this project.

## Conclusions

We conducted PPI in research by engaging people living with diabetes throughout the study, from identifying barriers to care to co-developing the mobile intervention and shaping study procedures. This paper describes a practical model of an structured co-creation process in an LMIC with the aim to generate digital health tools that are contextually relevant, acceptable, and more likely to be implemented successfully. An upcoming pilot study will be essential to evaluate usability, adherence, and early signals of impact, ultimately guiding future scale-up of the intervention developed for DFU-prevention. Other research teams using co-creation must balance participant expectations with study objectives by designing activities that are both informative for the project and meaningful to participants. Achieving this requires flexibility and adaptation in both the planning and delivery of workshops, allowing adjustments before and during sessions as needed.

## Supplementary Information

Below is the link to the electronic supplementary material.


Supplementary Material 1



Supplementary Material 2



Supplementary Material 3


## Data Availability

Data sharing is not applicable to this article as no datasets were generated or analysed during the current study. Instead, workshop reports were used to fill a matrix that allowed us to synthetize data. Workshop reports in Spanish could be shared upon reasonable request.

## Declarations

### Authors’ information (optional) AI usage

Large language models (LLMs) were used to translate the additional files from Spanish to English, the translations were then checked by one of the authors. Additionally, LLMs were used to improve flow in English.

## Abbreviations

CRONICAS: CRONICAS Center of Excellence in Chronic Diseases
DFU: Diabetic Foot Ulcer
mHealth: Mobile Health
HCPs: Healthcare professionals
PPI: Patient and Public Involvement
LMIC: Lower-Middle Income Country
PAR: Participatory Action Research
IRB: Institutional Review Board
NHR: National Institute for Health and Care Research
NAC: National Advisory Committee
LSHTM: London School of Hygiene & Tropical Medicine
UPCH: Cayetano Heredia Peruvian University
NCDs: Non-Communicable Diseases
