# Supplementary material for: The co-creation of Foot Selfie, a patient-centered mobile intervention to prevent diabetic foot ulcers
Source: BMC Health Serv Res. 2026 Jun 17;26:1201. doi: 10.1186/s12913-026-14718-5 (PMC13528000; doi:10.1186/s12913-026-14718-5)
Supplement: Supplementary file 1 [file 12913_2026_14718_MOESM1_ESM.docx]

**Additional file 1. Description of the Foot Selfie project and the app**

The Foot Selfie project has two work packages (WP). In WP1, the study 1) developed an artificial intelligence (AI) algorithm that will identify signs prior to ulcer onset using foot selfie images, 2) co-created of an intervention based on a mHealth tool for the prevention of DFU and the procedures to pilot test the intervention, and 3) pilot-tested the foot selfie mHealth tool to assess usability and accuracy of the AI algorithm. In WP2 is the program focused on engagement with stakeholders to explore how these interventions can be implemented in the Peruvian health system.

As part of aim 2) co-created of an intervention based on a mHealth tool for the prevention of DFU and the procedures to pilot test the intervention, we initially conducted two studies (not yet published) with the aim to better understand the challenges in preventing and managing DFU: 1) Patient journey mapping of diabetic foot prevention and care in a public healthcare facility. The goal was to explore the experiences of people with diabetes—including barriers and facilitators—when seeking care in the public health system before, during, and after developing DFU, and 2) a usability analysis of the app used to capture pictures of feet in WP1. These images were used to train an AI algorithm for early detection of pre-ulcerative lesions. This study aimed to explore barriers, facilitators, and motivators experienced by people with diabetes when taking the pictures at home.

The findings from both studies helped identify desirable features of an ideal mHealth app aimed at preventing DFU. With this information, a beta version of the mHealth app was developed and presented to the National Advisory Committee (NAC). This committee was established as a multisectoral space, composed of representatives from the Ministry of Health, universities, patient associations, and civil society organizations. Its purpose is to contribute to the design of sustainable strategies for the prevention and management of DFU in Peru, based on evidence and experience. A new beta version of the mHealth app was developed based on the feedback received by the NAC and used as a starting point for discussions in the workshops.

**Timeline of the co-creation component from the Foot Selfie Study**

**App Description**

The Foot Selfie application was initially developed in Android Studio using Kotlin (App versions 1–2) and subsequently migrated to Flutter (App versions 3–7). Version 1 was the only release that relied on simulated foot images; all later versions incorporated full functionality for image capture and uploading to a server, where an AI model for detecting pre-ulcerative characteristics on the plantar surface was deployed using Google Cloud Vertex AI.

During the co-creation process, the app’s front end was iteratively updated to incorporate feedback from workshop participants. In parallel, the team worked on improving the AI model for pre-ulcer detection. The workflow was designed so that enhancements to the AI model would not affect the app’s front-end functionality, enabling participant-driven modifications to be integrated seamlessly. Before processing, the app performs a basic image-quality check (orientation, sharpness, and illumination). On the server side, the pipeline normalizes the image, generates and refines masks, cleans contours, and computes simple metrics to estimate a risk level. The resulting image is displayed with an overlay to facilitate user interpretation.

Access controls ensure that each user can only view their own data, and all data is encrypted in transit. Data collection is minimized to meet privacy requirements. For performance optimization, the app reduces image resolution by 80%, compresses files prior to upload, and uses coroutines for asynchronous operations. When supported by the hardware, a Tesla T4 GPU is activated to accelerate inference.

With this architecture, we achieved a stable prototype suitable for field testing and ready for further iteration based on feedback from NAC.
