# Supplementary material for: The co-creation of Foot Selfie, a patient-centered mobile intervention to prevent diabetic foot ulcers
Source: BMC Health Serv Res. 2026 Jun 17;26:1201. doi: 10.1186/s12913-026-14718-5 (PMC13528000; doi:10.1186/s12913-026-14718-5)
Supplement: Supplementary file 2 [file 12913_2026_14718_MOESM2_ESM.docx]

**Additional file 2. Description of versions of the application**

| **Section** | **Initial version** | **Updated Version (7th)** | **Reasons for update** |
| --- | --- | --- | --- |
| Onboarding | This section was divided into interfaces that, through images, explained the objective of the application related to taking photographs and detecting early lesions. | This section has an animated video, which is shown only the first time you download the app. | Participants mentioned that a more dynamic and simple way to teach and motivate them was through animated videos that are easy to understand. |
| Registration | This section offered two alternatives for user registration:   1. Registration from scratch: personal data such as names and surnames, ID, gender, date of birth, height, weight, type of diabetes, region, health establishment, and emergency contact were requested. In addition, a password had to be created in compliance with security criteria and the option to recover password was shown. 2. Social Media Registration: Option to log in using Google or Facebook accounts. | This section offers two alternatives for user registration:   - Registration from scratch: only with cell phone number, password creation and password recovery option. - Social Media Registration: Option to log in using Google or Facebook accounts.   There is a checkbox to accept terms and conditions. | Participants mentioned that it would be easier to use their own phone numbers or the social media accounts already registered on their devices to access the app quickly without entering additional personal data. |
| Profile | A profile picture, registered email, buttons for: notifications, help center, my contacts, terms and conditions, log out appeared. | It was removed as a section but added as an interface that allows the creation of multiple users on the same device. Some of the data from the first version of the app was added in the "More" section. | Participants mentioned that having an interface that facilitates the use of the application in households with several people with diabetes and a single mobile phone would be beneficial. This function would also allow health professionals to create profiles for multiple patients. |
| Home | This section was divided into 4 sections:   1. General information: name, region, age, height, weight, and pre-set profile picture. 2. Explanatory message about the benefits of using the App. 3. "Your Last Checkups" option to review previous photos taken with the app. 4. Information section on diabetic foot and/or diabetes care and health in general. | This section is divided into 4 sections:   - Learn how to take photos: Watch the video tutorial on how to take photos. - Get to know what this app has to offer: Watch video onboarding - Get to know all the features: Interactive tour of the app. - Frequently Asked Questions: Provides technical support on the application.   It also has a floating Chat Bot-type messaging icon that will provide you with technical support on the application. | Participants mentioned the importance of prioritizing information related to the use of the application, photo capture, and questions regarding how to use the app. |
| Care | Was not present in the initial version of the app. | This section is divided into 3 sections:   - Videos: 3 preset videos on diabetes and diabetic foot. “Plus”, an extra button, to see more tips (educational videos and posters). - Alarm: Tool configured to encourage the use of the app for taking pictures. - Agenda: A tool created to provide people with a space where they can write down the dates of their appointments, medical exams and more. | Participants valued the idea of having a learning section on diabetes and diabetic foot from a comprehensive approach, identifying it as a current gap. Other features, such as the alarm and calendar, would serve as reminder tools. |
| Camera | This section was previously called "Add" and was divided into:   - Main interface: it was shown with a small frame, only buttons to take photos and reverse camera appeared. - Post-photo interface: a message on instructions for taking pictures, buttons to retake photos and continue. - Proper photo confirmation interface: Indicated if the photo had been taken correctly, buttons to view results and return to the main menu. - Results interface: Photo of the sole of the foot, message about the number and type of lesions identified, advice, reminder icon to schedule another photo and buttons to save results and return to the main menu. | This section is divided into:   - Main interface: The frame was enlarged to cover almost the entire screen of the cell phone, the buttons to take photos, reverse camera, gallery, flash and microphone were added. The microphone icon allows you to use a voice command ("take photo", "photo") to capture a picture without the need to press any button. - Interface after taking a picture: Indicates if the image is being processed properly and, in case the sole of the foot is not recognized, it has two buttons: "take another photo" and "see instructions". - Results interface: Indicates in the same photo of the sole of the foot, through colored segments to indicate where the lesion is. Then, it presents a table of the foot risk level with its respective alert, indication, and an informative video on how to proceed with this result. It also has two functionalities: share the result on your social networks and set the alarm for the next photo shot. | For participants, it is important to have multiple ways to take photos so they can try to do it on their own.  They also shared ideas about the results interface, noting how helpful it would be to include a color-coded risk level to guide appropriate actions, accompanied by a video featuring a medical professional who explains the type of pre-ulcerative lesion and what can be done about it. |
| Results | Was not present in the initial version of the app. | This section is divided into two:   - Progress Notification: Default messages according to the goal of taking photos per month. - Your last check-ups: Record of photographs of the soles of the feet from the first photo. - View Results Button: Displays a summary interface of results of your first and last photos in the 3-month time range of both soles of feet, as well as their risk levels to identify if there are changes in the results. | Participants recommended including a section that tracks their progress in taking photos and allows them to review and share their most recent results, particularly to support the identification of changes throughout their period of app use. |
| More | Was not present in the initial version of the app. | This section is divided into 7 topics:   - Diabetic foot clinic. - Diabetic foot organizations. - Edit Account - Terms & Conditions - Log off - Delete account - Change language | Participants expressed the need to learn more about diabetes prevention in their local areas and to be connected with relevant organizations. Therefore, we propose including a directory of diabetic foot clinics and organizations to facilitate access. They also highlighted the importance of providing clear options to recover passwords and to read privacy and data-use policies. Additionally, they suggested enabling the option to take photos with the phone even when there is no internet connection. |
