# Supplementary material for: The co-creation of Foot Selfie, a patient-centered mobile intervention to prevent diabetic foot ulcers
Source: BMC Health Serv Res. 2026 Jun 17;26:1201. doi: 10.1186/s12913-026-14718-5 (PMC13528000; doi:10.1186/s12913-026-14718-5)
Supplement: Supplementary file 3 [file 12913_2026_14718_MOESM3_ESM.docx]

**Additional file 2. Study procedures for the future pilot study**

| Stages | Description | Recommendations | Additions/modifications after the workshop |
| --- | --- | --- | --- |
| #1 | Recruitment of participants | - Strategic places for dissemination of the study: The explanation of the project should not be limited only to the doctor's office. Among the suggestions, the participants indicated that the waiting room is an ideal place to provide initial information, since it allows them to be informed while they wait to enter the consultation. Also, the use of public spaces should be included. - Use of didactic materials: During the workshop, the incorporation of visual resources to support the explanation (e.g., flipcharts, posters) was recommended. If possible, videos could be displayed on the doctors’ office TV. - Highlight the importance of self-care during the presentation: For the participants, it is important that, during the first approach, the team can raise awareness about the disease and the usefulness of the app, so they can encourage their participation in the pilot. - Include incentives for people to use the application: During the session, participants indicated that they can have incentives such as: (1) diabetic foot care kits (creams, gauze, soap, bandages, files, insoles, stockings, and glucometers and test strips), (2) economic incentives to support healthy eating for those who live in rural areas or their transfer. They also proposed that the incentives are personalized according to the need of each person. In addition, they mentioned that the distribution must oversee the project or some company | - The role of the healthcare professional is incorporated so that they provide information to potential participants who attend consultations. We did not perform open dissemination of the study because participants have specific characteristics (Grade 2 and 3 risk of ulceration). - Only one educational material on foot care is delivered and will be distributed through the healthcare professional (and not by the study personnel), to ensure sustainability. - Education and dissemination materials were developed: flipcharts, a diabetic foot flyer was made (10 commandments of diabetic foot care), and a video. - Monetary incentives (40PEN, ~10USD) will be provided for participants who require a transfer for the final interview. |
| #2 | Using Technology [App Installation, Familiarization] | - Identify the type of cell phone and participants' familiarity with its use to be considered eligible: During the session, participants and health personnel noted that many patients, especially older adults or people with conditions that make it difficult to understand, require a caregiver to be present during the use of the app. Thus, it was proposed to approach the potential participants and assess familiarity with cellphones or to directly approach family members (daughters, caregivers, etc.) to ensure that the participants have support when using the app. - Include practice materials on cell phone use: It was proposed to add some materials that will help participants support them when they forget how to use some functionalities of the app. | A tutorial is being designed for the use of the application. The application incorporates the section "know what the application offers you and learn to take photos". Here you will find all the information regarding the application and its functions. |
| #3 | App usage tracking | - Include materials to explain the contents of the application: During the session, participants proposed to include an interactive guide or flipchart to explain the step-by-step at the beginning, although this was not fully represented in the scene. - Include in the explanation: "what happens if you change cell phones?": The participants asked to add an associated answer to this question so that no concerns arise in new users when changing equipment or recovering them due to technical failures. - Incorporate a contact number in cases of technical support: This number will serve to settle concerns about problems with the camera or with the use of the application. - Reinforce the message that follow-up calls will be made. Participants proposed highlighting the message that follow-up messages will be made. These follow-ups should include reminders, greetings, or motivational phrases. | - There is a dataset with ID of each participant. It will serve as a backup. The dataset will be located on a save server. - A contact number will be available for inquiries. The WhatsApp channel will also be available for consultations. To complement the strategy: field staff will provide their institutional contact numbers. - No calls will be used but notifications will be sent once per week. There are 3 types of notifications: 1) for those who did not take a photo, 2) for those who took at least one photo, 3) for those who took more than 2. |
| #4 | Return of results, recommendations | - Return of printed and digital results: It was proposed that the return of results be carried out physically (brochures, videos, practical tips), virtual (report sent through WhatsApp). - Results accompanied by images of the first and last take: In the explanation of the graph of results, it was suggested to use the photographs from beginning to end to identify the changes that the person has had in their care. - Incorporate the personalized recommendation during the feedback of results: The proposal was made to include an emotional or motivational component in the feedback using messages of recognition for the effort made. | - The use of graphs to see the evolution of the lesions remains to be defined. An alternative proposal: foot insoles, indicating the percentage of the injury. Another: showing the caption (real photo). - It is incorporated into the report as a final message with the purpose of encouraging the care of their feet. |
